# Alveolus-like organoid from isolated tip epithelium of embryonic mouse lung

**DOI:** 10.1007/s13577-019-00236-6

**Published:** 2019-01-11

**Authors:** Yukihiro Seiji, Takaaki Ito, Yasuko Nakamura, Yuko Nakaishi-Fukuchi, Akira Matsuo, Naruki Sato, Hiroyuki Nogawa

**Affiliations:** 1 Department of Biology, Chiba University Graduate School of Science, Chiba, Japan; 2 Department of Pathology and Experimental Medicine, Kumamoto University Graduate School of Medical Sciences, 1-1-1 Honjo, Chuo-ku, Kumamoto, 860-8556 Japan

**Keywords:** Alveolus, Organoid, Tip epithelium, Embryonic mouse lung, Fibroblast growth factor 7

## Abstract

Embryonic lungs were obtained from embryonic day 13.5 ICR mice. The lung-tip epithelium isolated using dispase treatment was embedded in low-growth factor Matrigel, cultured in DMEM/F12 medium containing 0.1% bovine serum albumin, supplemented with insulin, transferrin, and selenium (ITS), with or without fibroblast growth factor 7 (FGF7), and were observed for 14 days. With the addition of FGF7, the tip epithelium grew to form a cyst by culture day 7. Then, tubular tufts-like alveolus appeared around the cyst surface. Reverse transcription-polymerase chain reaction revealed that, with the addition of FGF7, the cultured lung explants expressed alveolar-type 1 cell markers, such as *HopX* and *Aquaporin5*, and type 2 cell markers, such as *Lamp3* and *Surfactant apoproteins* (*Sftp*) *C* and *D*. Paraffin-embedded sections were stained with hematoxylin and eosin, and alveolar structures at culture day 14 were composed of squamous and cuboidal epithelial cells. Immunohistochemical studies showed that the squamous epithelial cells were positive for HopX, and the cuboidal epithelial cells were positive for pro-SftpC. Furthermore, transmission electron microscopic observation confirmed that the squamous epithelial cells were alveolar-type 1 cells and the cuboidal cells were type 2 cells, because they had many lamellar inclusion bodies. Embryonic lung-tip epithelium forms an alveolus-like organoid through the self organization with the aid of Matrigel, ITS, and FGF7. This method to make alveolus-like organoid in vitro is easy, reproducible, and economical. This method could have potential to solve many issues in alveolar epithelial cells in normal and pathological conditions.

## Introduction

In the respiratory system, the developing embryonic lung epithelium shows dramatic alterations associated with branching morphogenesis, cellular differentiation, and functional maturation. Development of the lung proceeds in a proximal to distal direction, starting in the trachea and progressing to include lobar bronchi, bronchioles, and alveoli [1–3]. The distal end of the lung forms a delicate system of alveoli, and alveoli are the critical air spaces facilitating gas exchange, the principal function of the lung. During the pseudoglandular stage of the developing lung [embryonic day (E) 10.5–16.5], sequences of branching modes including domain branching, planar bifurcation, and orthogonal bifurcation make a developed branched organ [3]. During the canalicular stage (E16.5–17.5), undifferentiated epithelia continue to branch and make terminal sacs. After the terminal sac stage (E17.5 and thereafter), the distal tip epithelium gives rise to alveolar epithelial cells [4]. Alveolar sacs are lined by alveolar-type 1 cells and alveolar-type 2 cells; alveolar-type 1 cells mediate gas exchange, and alveolar-type 2 cells secrete surfactant, which prevents the collapse of the alveolar space. During development, alveolar-type 1 cells and type 2 cells arise directly from a bipotent progenitor [5].

The process of lung organogenesis seems to need complex and well-organized cell signaling systems, which are regulated by various transcription factors. Cell signaling and transcriptional networks could drive cell proliferation, branching morphogenesis, differentiation, and alveolization [6]. Many transcription factors are active in endodermally originated cells of developing lung epithelium, including thyroid transcription factor-1 (TTF1), β-catenin, Forkhead orthologs (FOX), GATA, Sox, and ETS family members, are necessary to establish normal lung morphogenesis and cell differentiation [6]. Several growth factors have been implicated in regulating lung morphogenesis, including fibroblast growth factors (FGFs), Wnts, bone morphogenetic proteins, and sonic hedgehog. FGF signaling is an essential system regulating morphogenesis, maintaining progenitor populations, epithelial and mesenchymal patterning, and differentiation [7]. FGF10 and its receptor, FGF receptor2b (FGFR2b), are necessary for lung morphogenesis [8–10]. FGF7 is expressed in the mouse developing lung mesenchymal tissues [11, 12]. FGF7 shares FGFR2b with FGF10 as a specific receptor [13], but recombinant FGF7 and FGF10 induce different epithelial reactions in cultured embryonic lung explants [14–16]. Different epithelial responses to FGF7 versus FGF10 via FGFR2b could be explained by the different active PI3K-AKT pathway signaling durations; sustained signaling induced by FGF10 could be related to epithelial migration, whereas transient signaling induced by FGF7 influences cell proliferation [7].

Embryonic isolated epithelial explants can survive and differentiate with an extracellular matrix such as Matrigel substratum, and growth factors can modify branching morphogenesis [17–20]. Lung organoid refers to self-assembling structures generated from lung epithelial progenitor cells cultured with 3D support with or without mesenchymal supporting cells [21]. Establishment of lung organoid has great promise to answer various questions on the cellular and molecular mechanisms of lung development and disease mechanisms, and to afford an important tool for surveying various chemicals and therapeutic drugs. It is not achievable to recapitulate the complete lung structure, although the recent studies involving lung organoids have been well designed with consideration of the molecular mechanisms of lung development [16, 22–24].

In the present study, we used isolated mouse lung-tip epithelia cultured in a serum-free medium containing a supplement containing insulin, transferrin, and selenium (ITS) and FGF7. We then examined culture explants using immunohistochemical, electron microscopic, and reverse transcription-polymerase chain reaction (RT-PCR) analyses. The explants grew and formed alveolus-like organoid structures containing alveolar-type 1 and type 2 cells. Recent studies by Miller et al. [16, 25] showed a similar explant culture experiment using isolated epithelium of embryonic mouse lung treated with FGF7, FGF10, BMP4, retinoic acid, and a GSKβ3 inhibitor to identify conditions that maintain epithelial tip progenitors in vitro and further apply lung bud tip progenitor cells derived from human pluripotent stem cells. We established a simple, easy, and reproducible method to make alveolus-like organoids, and here introduced this method for future studies in the field of lung biology and medicine.

## Materials and methods

### Lung rudiments

**Fig. 1:**
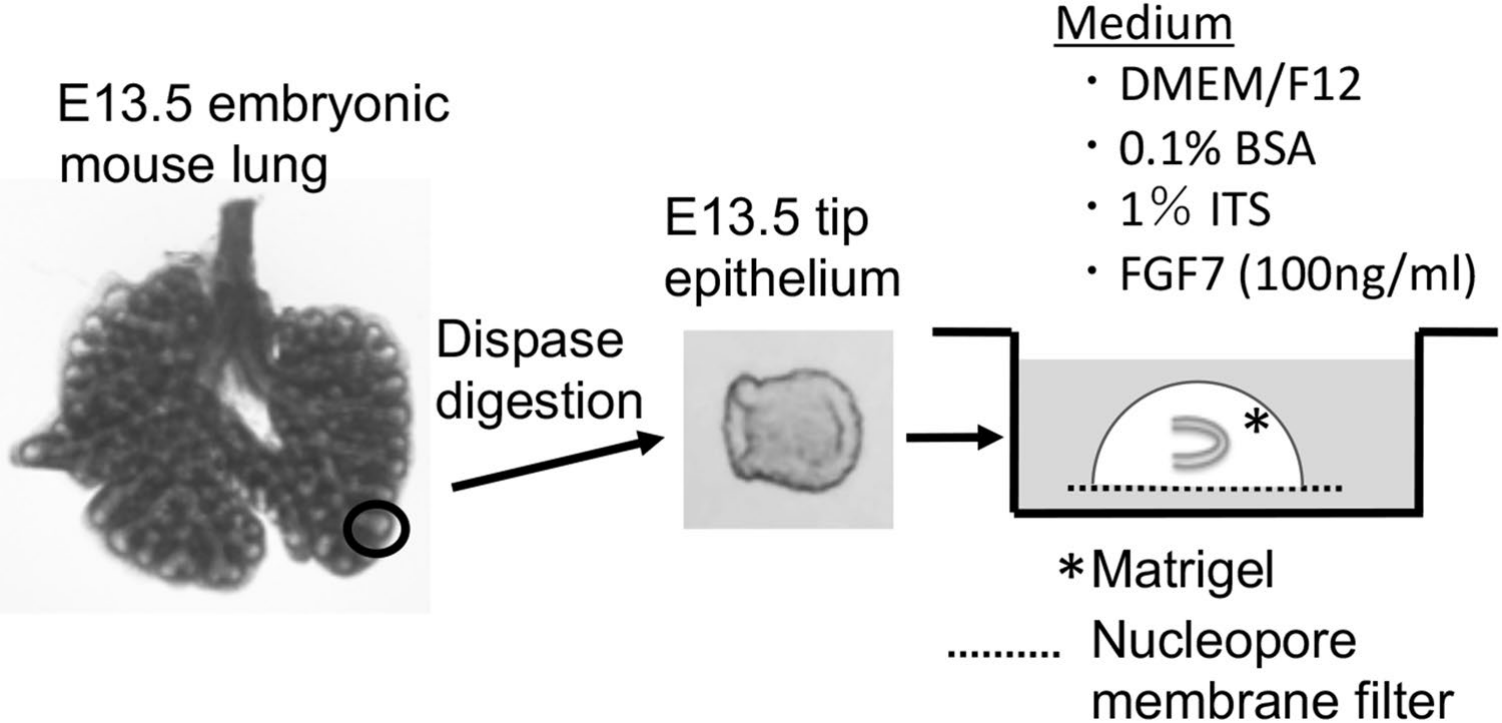
Experimental design. The lung rudiments were obtained from E13.5 mice, and the tip epithelium was obtained by digestion with dispase. The E13.5 isolated tip epithelium was embedded in growth factor-reduced Matrigel on a nucleopore membrane filter, and cultured in DMEM/F-12 medium with ITS (1%) and FGF7 (100 ng/ml)

ICR mice were mated at night, and the day of the discovery of a vaginal plug was designated as day 0. Lung rudiments were isolated from E13.5 embryos in Hanks’ balanced salt solution (HBSS). E13.5 rudiments were treated with dispase (1000 protease units/ml in HBSS; Godo Shusei Co., Tokyo, Japan) at 37 °C for 40 min, and distal epithelial tip (E13.5 tip) isolated from right caudal or left lobes was separated from mesenchymal tissues with fine forceps (Fig. 1). E13.5 tips were used in culture experiments and RT-PCR analyses.

### Epithelium-alone culture

Epithelium-alone cultures were conducted according to the methods of our previous studies with some modifications [17, 26] (Fig. 1). Two base gels were made with 3 µL Matrigel (growth factor-reduced; BD Biosciences, Bedford, MA, USA) on a half-cut nucleopore polycarbonate membrane filter (diameter 13 mm, pore size 0.1 µm; Whatman, Kent, UK) in the inner well of a Falcon 353708 dish (BD Biosciences, Bedford, MA, USA). E13.5 tips were placed on base gel and then covered with 5 µL Matrigel. After gelling, 500 µL medium was poured into the well, in which the E13.5 tip-Matrigel-filter was submerged in medium, which was detached from the bottom of the dish. Each filter containing one or two E13.5 tips was cultured at 37.5 °C in 5% CO_2_/air. The basal medium was composed of Dulbecco’s modified Eagle’s medium and nutrient mixture Ham’s F-12 (1:1; Invitrogen, Carlsbad, CA, USA) supplemented with 0.1% bovine serum albumin (fatty acid-free; Sigma-Aldrich, St. Louis, MO, USA), and 1% Insulin–Transferrin–Selenium-G-Supplement (ITS, Invitrogen). As an additional growth factor, FGF7 (human recombinant, animal-derived-free; PeproTech, London, UK) was added to the culture medium at a final concentrations of 100 ng/ml. All culture experiments were repeated at least three times until the number of explants in the same culture conditions increased to at least 6.

### Histological techniques

Cultured epithelium was fixed in Bouin’s fluid, dehydrated, and embedded in paraffin. Sections were prepared at 5 µm thickness, rehydrated, and stained with hematoxylin and eosin.

### RT-PCR

Total RNA was isolated from E13.5 tip epithelia, and cultured tip epithelia at 3, 7, and 14 days using TRIzol reagent (Invitrogen), and cDNAs were synthesized using a SuperScript First-Strand Synthesis System for RT-PCR (Invitrogen). PCR amplifications were performed using AmpliTaq Gold 360 PCR Master Mix (Applied Biosystems, Branchburg, NJ, USA) with the following parameters; 30, 35, and 40 cycles of 94 °C for 30 s, annealing temperatures suitable for each primer set (54–56 °C) for 30 s, and 72 °C for 60 s after the initial activation of the Taq enzyme at 94 °C for 10 min. The primer sets are listed in Table 1. The PCR products were separated by 1.7% agarose gel electrophoresis and visualized by ethidium bromide staining. The quantity of cDNA in each sample was measured using ImageJ and was normalized against β-actin mRNA expression. The experiments were repeated three times independently.

**Table 1:** xxx

	Accession no.	Annealing temperatures	Forward	Reverse
*Pdpn*	NM_010329.2	54	5′-GAAACGCAGACAACAGATAAG-3′	5′-ACCCATGGTTACAGTTGCTA-3′
*Hopx*	NM_001159900.1	56	5′-CTCCATCCTTAGTCAGACG-3′	5′-CAGCCAAGCCATCACTTTAC-3′
*Aqp5*	NM_009701.4	56	5′-ATCATAGAGAGGAGCGGAAG-3′	5′-CACTCGACGAACCATCTATC-3′
*SftpC*	NM_011359.2	54	5′-AGATGGTCCTTGAGATGAGC-3′	5′-TTACAGACTTCCACCGGTTT-3′
*Lamp3*	NM_177356.3	56	5′-CTACAGAAGACTCAAGCACAC-3′	5′-GACAGAGTTGGCCTCTGATT-3′
*SftpD*	NM_009160.2	56	5′-CAAAGGGAGAACGTGGACTA-3′	5′-CTGATAGTGGGAGAAGGCAA-3′
*Scgb1a1*	NM_011681.2	54	5′-AAGATCGCCATCACAATCAC-3′	5′-CGCAGTTTATTGCAAAGAGG-3′
*Foxj1*	NM_008240.3	54	5′-GCAGAATGGAAGTGAGTGTT-3′	5′-ATAGTCCACGTCGTCAGG-3′
*Fgfr2*	NM_201601.2	54	5′-GAATCCAACGTCCACAATGA-3′	5′-ATCTCCGTCACATTGAACAG-3′
*Sox2*	NM_011443.4	56	5′-ATGATCAGCATGTACCTCCC-3′	5′-GCCTAACGTACCACTAGAAC-3′
*Sox9*	NM_011448.4	54	5′-CCAACATTGAGACCTTCGAC-3′	5′-TTCTGATGGTCAGCGTAGT-3′
*β-actin*	NM_007393.5	56	5′-TCAGAAGGACTCCTATGTGG-3′	5′-TCTCTTTGATGTCACGCACG-3′

The data were analyzed by one-way analysis of variance followed by multiple comparison analysis using Ryan’s test [27, 28].

### Immunohistochemistry

The explants at culture day 14 were fixed with phosphate-buffered 4% paraformaldehyde solution for 24 h, and embedded in paraffin. These sections were rehydrated with xylene and graded ethanol solutions. After being treated with 0.3% hydrogen peroxide, the sections were heated at 95 °C for 40 min in antigen retrieval solution (pH 8, Nichirei, Tokyo, Japan). After being treated with skimmed milk solution for 10 min, the sections were treated with mouse anti-HopX antibody (Santa Cruz biotechnologies, Santa Cruz, CA) and rabbit anti-pro-SftpC antibody (Chemicon, Temecula, CA) overnight at 4 °C. After washing, the sections were treated with anti-mouse or anti-rabbit IgG conjugated with the HRP polymer (DAKO, Glostrup, Denmark) for 1 h at room temperature. The sections were further treated with diaminobenzidine-hydrogen peroxide solution, and counterstained with hematoxylin.

### Electron microscopy

The explants at culture day 14 were fixed with cacodylate-buffered 2% glutaraldehyde solution for 24 h, and, after rinsing, post-fixed with 1% osmium tetroxide. The explants were stained en bloc with uranyl acetate to preserve lamellar inclusion bodies [29], dehydrated with a graded ethanol, and embedded in Epon–Araldite mixture. Thick sections were stained with 1% toluidine blue before making ultra-thin sections. The ultra-thin sections were examined with a Hitachi H7500 electron microscope.

## Results

### Morphogenesis of embryonic lung-tip epithelium in Matrigel with FGF7

**Fig. 2:**
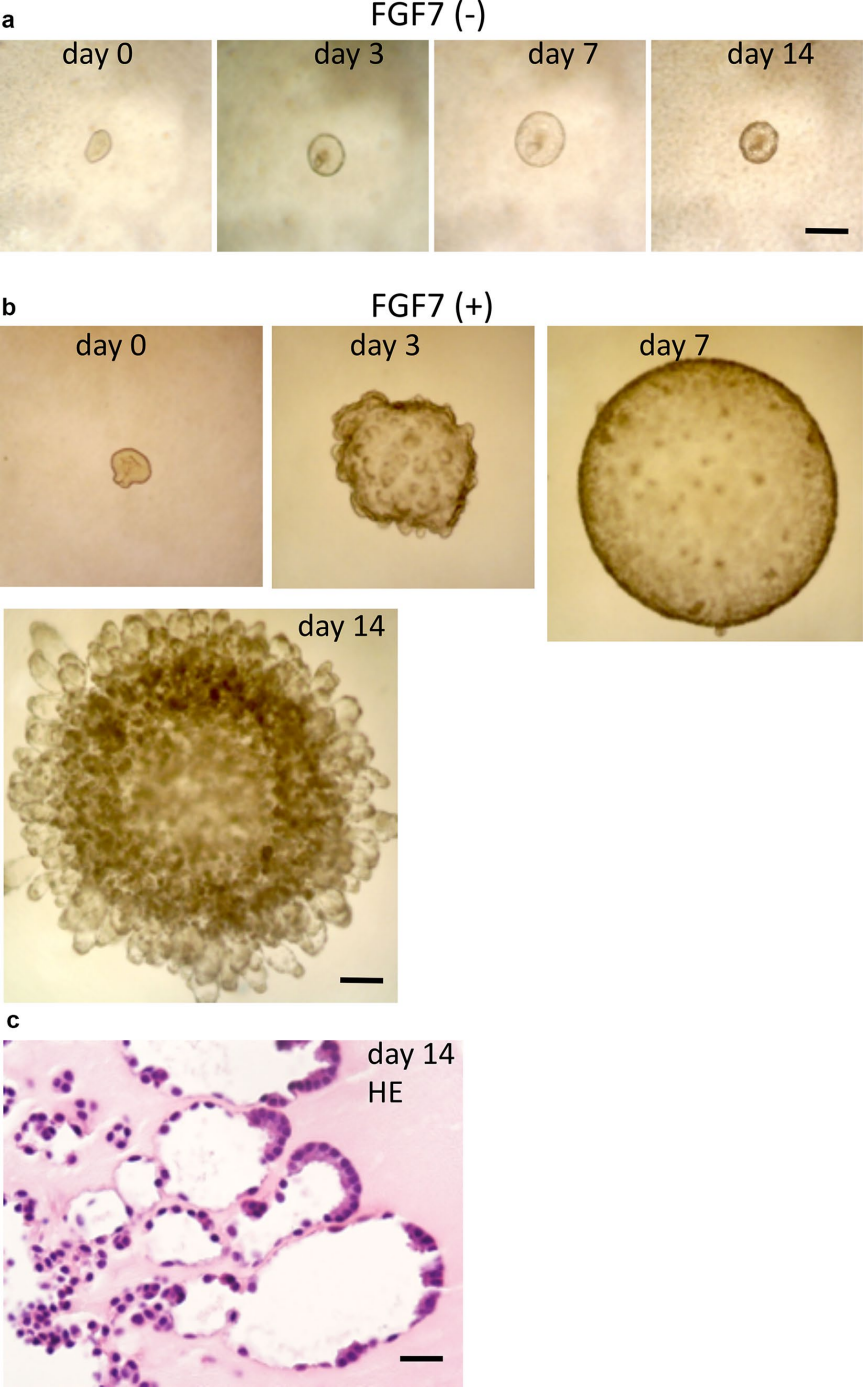
Morphological changes in cultured E13.5 tip epithelium. **a** Without FGF7, the isolated epithelial tip does not show remarkable morphological changes. Bar = 200 µm. **b** With the addition of FGF7 (100 ng/ml), the epithelial tip grows and dilates to form a cyst up to culture day 7. Then, many buds appear from the surface of the cyst and produce many alveolus-like tufts by culture day 14. Bar = 200 µm. **c** Histology of the alveolus-like organoid at culture day 14. The luminal area of the cyst is irregularly lined by cuboid epithelial cells, and the tufts are composed of flat and cuboidal cells. Cuboidal cells are often located peripherally in the tuft. Hematoxylin and eosin staining (HE). Bar = 20 µm

The lung epithelial tips from E13.5 mice were covered with Matrigel and cultured in DMEM/F12 medium containing ITS with or without FGF7. Without FGF7, isolated tip epithelia formed small cysts, and were able to survive until culture day 14, but did not show notable morphological changes throughout the observation period (Fig. 2a). With the addition of FGF7 (100 ng/ml), the epithelial tip grew to form a large cyst around 1 mm in diameter by culture day 7, and then, numerous buds occurred radially throughout the cystic surface and formed many alveolus-like tufts (Fig. 2b). Light microscopic observation of the explants cultured for 14 days showed that the luminal area of the center of the cyst was irregularly lined by cuboid epithelial cells, and the tufts were composed of two types of epithelial cells, squamous epithelial cells and cuboidal cells, located at the periphery of the tufts (Fig. 2c).

### Modulation of mRNAs of alveolar cells in cultured lung epithelial tips

**Fig. 3:**
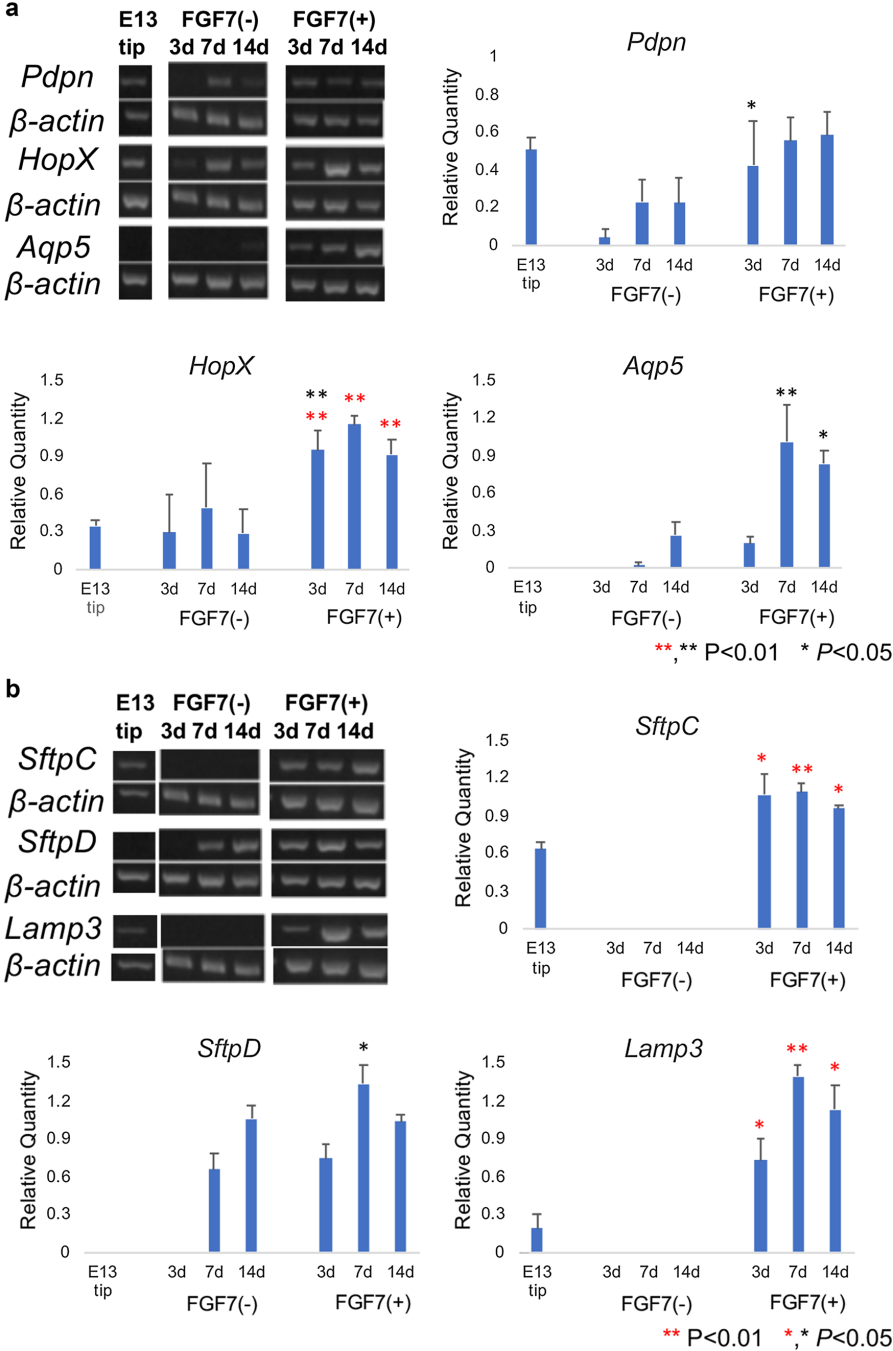

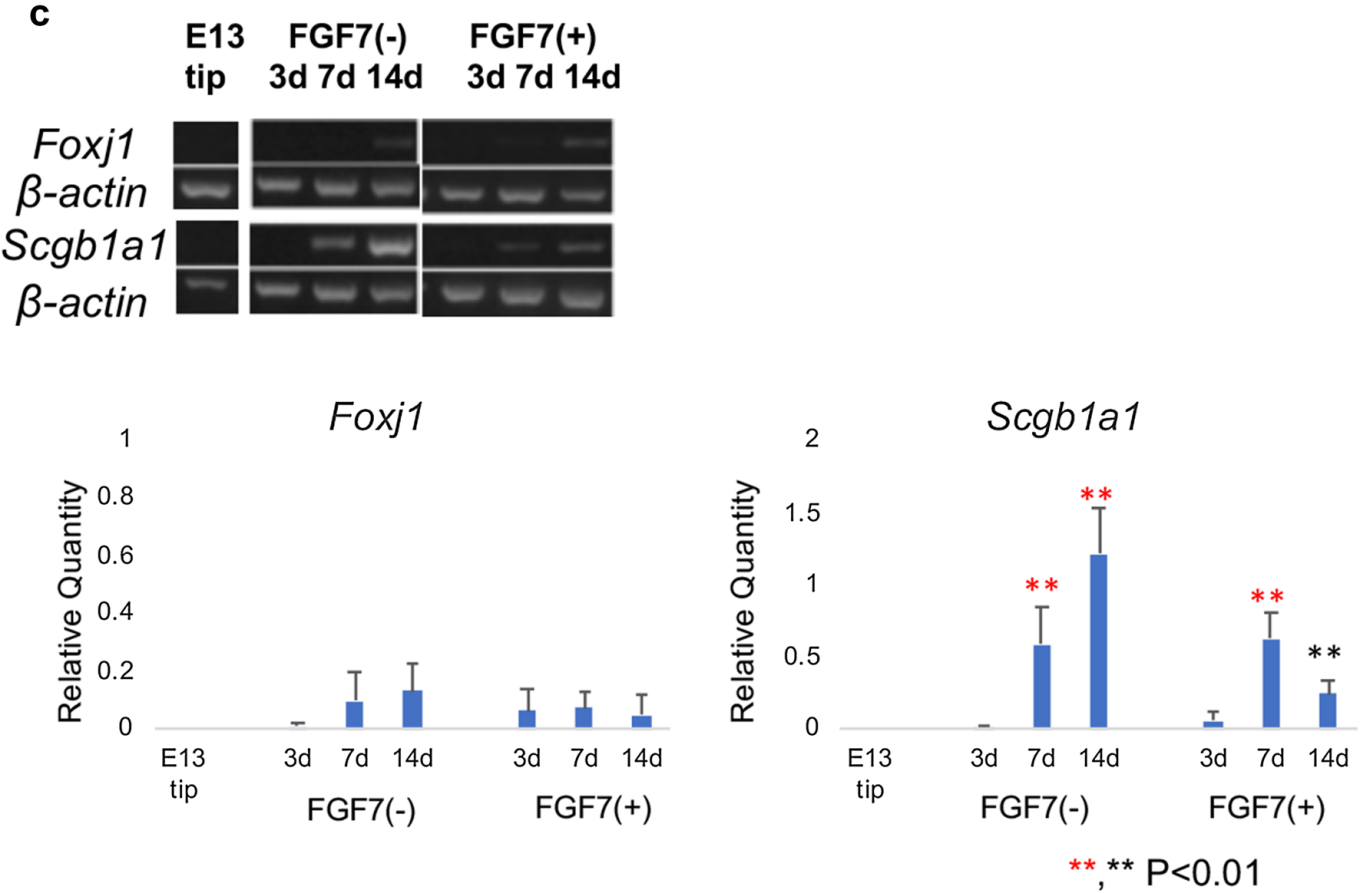
Modulation of mRNA expression of **a** alveolar-type 1 cell-specific, **b** alveolar-type 2 cell-specific, and **c** bronchiolar cell-specific molecules in tip epithelium of E13.5 mouse lungs (E13 tip) and their explants at culture day 14. **a** Regarding alveolar-type 1 cell-specific molecules, Pdpn, HopX, and Aqp5 mRNAs are examined by RT-PCR, and their values are corrected using the value of β-actin mRNA. Without treatment with FGF7, there are no remarkable changes in their expression except for a mild increase in *Aqp5* at culture day 14. With treatment with FGF7, *HopX* and *Aqp5* levels increase with culture. **b** Regarding alveolar-type 2 cell-specific molecules, SftpC, Lamp3, and SftpD mRNAs are examined by RT-PCR, and their values are corrected using the value of β-actin mRNA. Without FGF7 treatment, no remarkable changes in the expression of *SftpC* and *Lamp3* are seen, but SftpD mRNA increases slightly at culture day 14. With FGF7 treatment, the mRNAs levels of *SftpC, Lamp3*, and *SftpD* evidently increase at culture day 3. **c** Regarding bronchiolar cell-specific molecules, FoxJ1 mRNA, a ciliated cell-specific molecule, is not altered during the observation period either with or without FGF7 treatment. Scgb1a1 mRNA, a club cell-specific molecule, is increased slightly at culture day 7 either with or without FGF7. Statistical significances are indicated by asterisks: red indicates significance between the values of the E13.5 epithelial tip (E13 tip) and explants cultured without FGF7 (−) or with FGF7 (+), and black indicates significance between the values of the explants cultured without FGF7 (FGF7 (−)) or with FGF7 (FGF7 (+)) for the same culture periods

Without treatment with FGF7, the lung epithelial tip did not show remarkable alterations in the expression levels of molecules specific to alveolar-type 1 and type 2 epithelial cells, except for increased expression of Aqp5, SftpD, and Scgb1a1 mRNAs in explants after 14 days (Fig. 3a–c). With treatment with FGF7, mRNAs specific for alveolar-type 1 epithelial cells, such as *HopX* and *Aqp5*, increased significantly after culturing for 3 or 7 days (Fig. 3a), and mRNAs for alveolar-type 2 cells, such as *SftpC, SftpD*, and *Lamp3*, showed significantly increased expression after culturing for 3 days (Fig. 3b). In comparison with the alveolar epithelial cell markers, modulation in expression of the bronchiolar epithelial cell markers was modest. *FoxJ1*, a ciliated cell marker, was not expressed throughout the observation period, and *Scgb1a1*, a Club cell marker, showed mildly increased expression at culture day 7 (Fig. 3c).

**Fig. 4:**
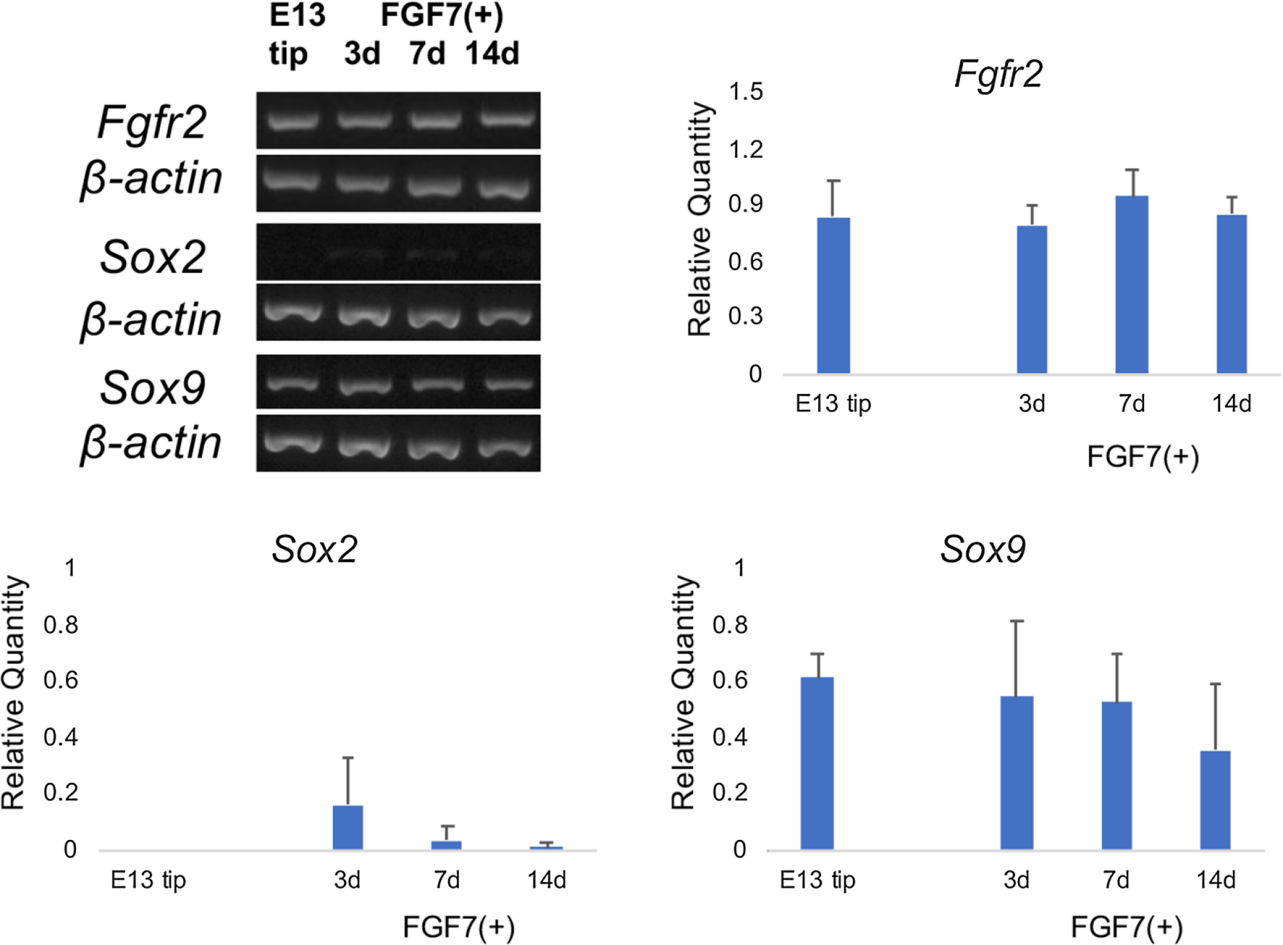
Effects of FGF7 on expression on FGFR2, Sox2, and Sox9 mRNA levels. Regarding the expression of FGFR2 mRNA, lung-tip epithelium from E13.5 mouse has FGFR2 mRNA, and it expression continues throughout the culture period. Sox2 mRNA is not detected in E13.5 tip epithelium (E13 tip) before and after culturing. Sox9 mRNA is detected in E13.5 tip epithelium (E13 tip); its expression is detected during the observation period

Besides epithelial-specific molecules, the expression levels of FGFR2, Sox2, and Sox9 mRNAs were examined as FGFR2 is the specific receptor for FGF7 [13] and Sox9 regulates alveolar epithelial development [30, 31]. FGFR2 mRNA expression was detected in E13.5 tip epithelium, and levels were constant throughout the culture period (Fig. 4). In E13.5 tip epithelium, Sox2 mRNA was not detected, and mildly increased with culture with FGF7 (Fig. 4). Meanwhile, Sox9 mRNA was present in the E13.5 tip epithelium, and continued to be present throughout the observation period (Fig. 4).

### Immunohistochemistry of alveolus-like organoid

**Fig. 5:**
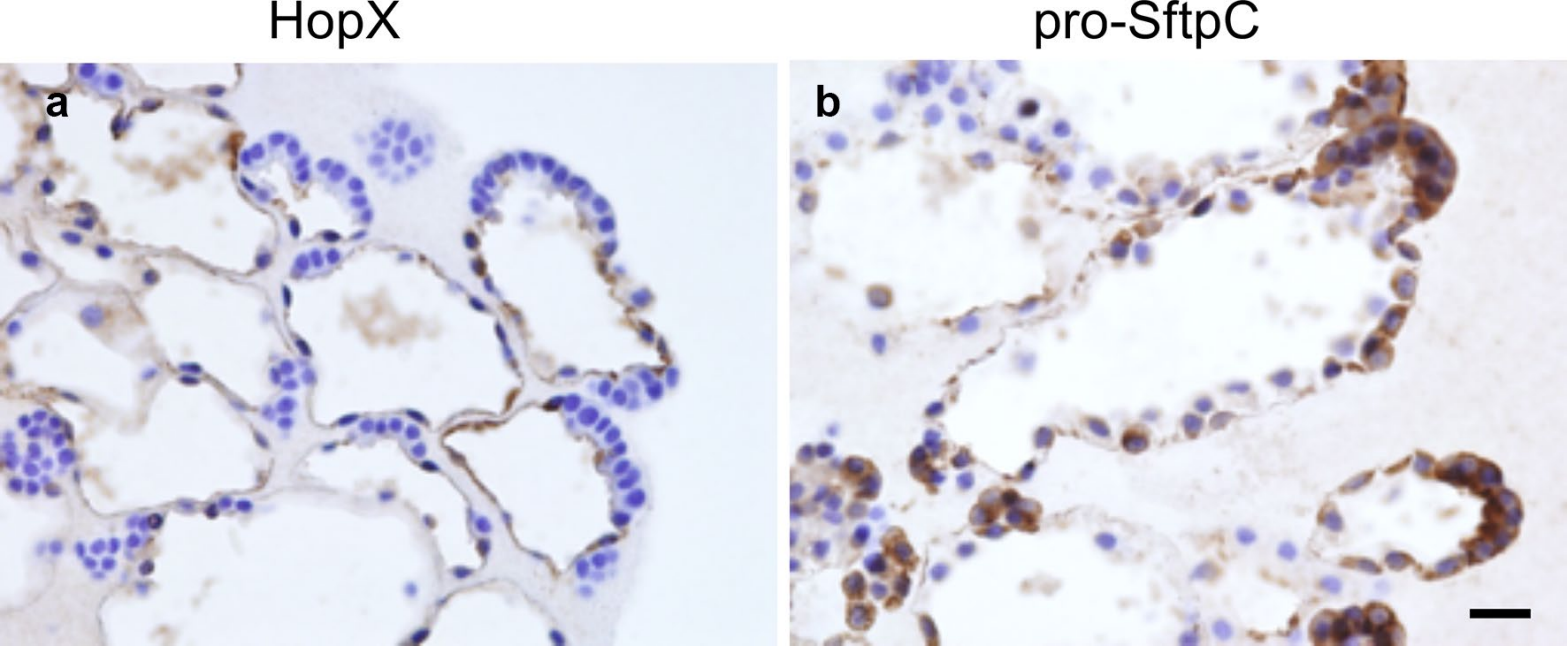
Immunostaining for alveolus-like organoid from tip epithelium culture with FGF7 for 14 days. **a** HopX, an alveolar-type 1 cell-specific protein, is positive in the squamous epithelium lining radiated growing tufts. **b** Pro-SftpC, an alveolar-type 2 cell-specific protein, is positive in the cytoplasm of the cuboidal cells lining the periphery of the tuft. Bar = 20 µm

The explants of the cultured e13.5 tip epithelium at culture day 14 with FGF7 were immunohistochemically examined for HopX, an alveolar-type 1 cell-specific protein, and for pro-SftpC, a type 2 cell-specific protein. In the central areas of the cysts, cuboidal cells were mixed with HopX-positive cells and pro-SftpC-positive cells. In the alveolus-like tuft areas of the cysts, cell surfaces of the squamous epithelial cells were positive for HopX (Fig. 5), and cuboidal epithelial cells at the periphery of the tufts showed positive cytoplasmic staining for pro-SftpC (Fig. 5). Occasionally, cell surfaces of the squamous epithelial cells were stained for pro-SftpC (Fig. 5).

### Electron microscopic observation of the alveolus-like organoid

**Fig. 6:**
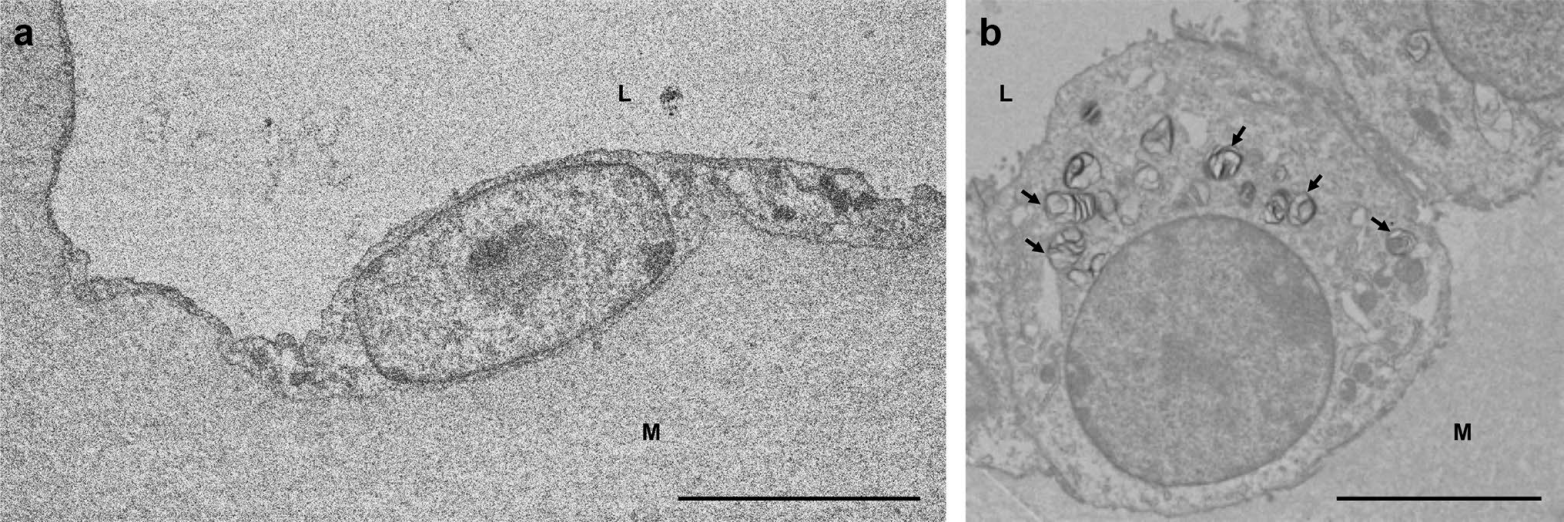
Transmission electron microscopic observation of cells lining the alveolus-like organoid from E13.5 tip epithelium cultured with FGF7 for 14 days. The alveolus-like structure is composed of squamous and cuboidal epithelial cells. L: lumen of the alveolus-like organoid. M: subepithelial matrix. **a** A squamous epithelial cell with thin cytoplasm is not well developed with organelles, and appears to be alveolar-type 1 cell. Bar = 5 µm. **b** A cuboidal cell has many lamellar inclusion bodies (arrows) and is considered as an alveolar-type 2 cell. Bar = 10 µm

Light microscopically, toluidine staining showed the alveolus-like tufts seen in the explants cultured for 14 days with FGF7 which were composed with squamous and cuboidal epithelial cells. Granules were present in the cytoplasm of the cuboidal cells and tuft lumens (data not shown). The alveolus-like tufts were observed using transmission microscopy. The squamous epithelial cells had a few organelles, and their extended and thin cytoplasm lined the lumen (Fig. 6a), which suggests that the squamous cells appear to be alveolar-type 1 cells. The cuboidal cells had microvilli on the luminal surface and many organelles as well as lamellar inclusion bodies in the cytoplasm (Fig. 6b), which suggests that the cuboidal cells appear to be alveolar-type 2 cells.

## Discussion

In the present study, we have described an easy, reliable, and economical method to make alveolus-like organoids from isolated epithelium of embryonic mouse lung tip in serum-free condition using Matrigel, ITS supplement, and FGF7. With this method, the isolated tip epithelia grow to form a cyst and produce many radial tufts mimicking alveolus-like structures, which are composed of alveolar-type 1 and type 2 cells. We used lung-tip epithelium obtained from E13.5 mice. During the developmental period, undifferentiated epithelial progenitors are present at the distal tips of the branching lung epithelia. The distal tip epithelial cells have high proliferative activity, and express transcription factors such as Sox9, Nmyc, and Id2 [4, 30–32]. Rawlins et al. [4] analyzed *Id2-CreER*^T2^ knock-in mice to trace the fate of distal tip epithelial cells during either the pseudoglandular and canalicular stages of development, and revealed that Id2-positive tip epithelial cells at E10.5–12.5 contributed to both the bronchiolar and alveolar cells, and that Id2-positive tip epithelial cells at E16.5 became alveolar epithelial cells. Our immunohistochemical and electron microscopic studies revealed that alveolus-like structures are composed of alveolar-type 1 and type 2 cells. This was supported by RT-PCR studies, in which the lung-tip epithelia showed positive reactions for alveolar-type 1 marker molecules, such as HopX and Aqp5, and also for alveolar-type 2 marker molecules, such as SftpC, SftpD, and Lamp3. However, there were no prominent reactions for bronchiolar epithelial cell marker molecules, such as Scgb1a1 and FoxJ1, throughout the culture period. In addition, at the same time, our RT-PCR analysis of E13.5 tip epithelia of mouse lungs showed high expression levels of Sox9 mRNA and low levels of Sox2 mRNA, and this expression pattern continued throughout the culture period. Thus, our alveolus-like organoid was composed mostly of alveolar epithelial cells, which could be attributed to the tip epithelium at E13.5 should be composed of alveolar epithelial cell-committed progenitor cells, and our culture methods could support Sox9 expression.

During the culture period, lung-tip epithelial explants appeared to grow through two stages and to form alveolus-like organoids; the first stage showed simple growth accompanying cyst formation, and the second stage involved radial growth with many alveolus-like tufts forming, although treatment was constant throughout the observation period. Actually, some alveolar cell markers, such as HopX, Aqp5, Lamp3, and SftpD, were present at relatively low levels at culture day 3, but levels were high after culture day 7, which seemed in accordance with the morphological changes. It was conceivable that some critical molecular events might occur between the two steps. During the observation period, the Sox2/Sox9 balance was similar and FGFR2 was not modulated, which suggested that the intracellular molecular environment of the transcription factor network or signaling pathways might change during the culture period. Alternatively, it could be suspected that surfactant secretion from maturing alveolar-type 2 cells could help extension of the tufts.

Miller et al. [16, 25] reported a similar, but more sophisticated organoid culture study using tip epithelium from E13.5, and they examined the effects of FGF7, FGF10, CHIR, and RA on growth of the distal tip epithelium and transcription factors such as Nmyc, Sox2, Sox9, and Id2 to elucidate the mechanisms of lung development. They reported the effects of FGF7 alone on morphogenesis, proliferation, and expression of transcription factors, and some epithelial markers. According to their report, FGF7 alone promoted growth, expansion, and survival of isolated buds up to 2 weeks, which was similar to the observation in our study, but the organoid structure formed was different. In our study, distal tip epithelium formed a cystic sphere at first and then produced many radial tufts mimicking alveolar structures, but the organoid of Miller et al. formed branches early in the culture. Both studies showed that FGF7 maintains the expression of Sox9 and suppresses the expression of Sox2, and it induced marker molecules for alveolar-type 1 and type 2 cells. However, in spite of increased Scgb1a1 mRNA in the study by Miller et al. [16, 25], a small increase in the expression of the Club cell marker molecule was detected in the present study (Fig. 3c). The different results in the two studies could be attributed to the different concentrations of FGF7 (100 ng/ml (our study) vs. 10 ng/ml [16, 25]) and the supplements which we used. We supplemented the medium with ITS (containing insulin, transferrin, and selenium), which is universally used in various cell cultures in place of bovine serum [33].

In the present study, we demonstrated that tip epithelium from embryonic mouse lung develops into alveolus-like organoids after cultivation for 14 days with ITS supplement and FGF7. This alveolus-like organoid could constitute an excellent experimental model to clarify the molecular mechanisms of alveologenesis and alveolar cell-fate determination using mice with mutated genes of interest and genome editing using CRSPR-Cas9 knock-in mice [34]. Moreover, co-culture with endothelial cells might develop into functional alveoli with ability for gas exchange in vitro. In addition, this alveolus-like organoid could be useful in studying regeneration and tumorigenesis processes and for testing the effectiveness of chemicals. Thus, this alveolus-like organoid model has potential for the examination of various issues in alveolar epithelial cells in both normal and pathological conditions.

## References

[1] Sorokin S, De Haan RL, Urpsrung H (1965). Recent work on developing lungs. Organogenesis.

[2] Cardoso WV, Lu J (2006). Regulation of early lung morphogenesis: questions, facts and controversies. Development.

[3] Metzger RJ, Klein OD, Martin GR, Krasnow MA (2008). The branching program of mouse lung development. Nature.

[4] Rawlins EL, Clark CP, Xue Y, Hogan BLM (2009). The Id2^+^ distal tip lung epithelium contains individual multipotent embryonic progenitor cells. Development.

[5] Desai TJ, Brownfield DG, Krasnow MA (2014). Alveolar progenitor and stem cells in lung development, renewal and cancer. Nature.

[6] Maeda Y, Dave V, Whitsett JA (2007). Transcriptional control of lung morphogenesis. Physiol Rev.

[7] Volckaert T, De Langhe SP (2015). Wnt and FGF mediated epithelial mesenchymal crosstalk during lung development. Dev Dyn.

[8] Min H, Danikenko DM, Scully SA (1998). Fgf-10 is reguired for both limb and lung development and exhibits striking functional similarity to Drosophila branchless. Genes Dev.

[9] Sekine K, Ohuchi H, Fujiwara M (1999). Fgf10 is essential for limb and lung formation. Nat Genet.

[10] De Moerlooze L, Spencer-Dane B, Revest JM, Hajihosseini M, Rosewell I, Diclspn C (2000). An important role for the IIIb isoform of fibroblast growth factor receptor 2 (FGFR2) in mesenchymal-epithelial signaling during mouse organogenesis. Development.

[11] Mason IJ, Fuller-Pace F, Smith R, Dickson C (1994). FGF-7 (keratinocyte growth factor) expression during mouse development suggests roles in myogenesis, forebrain regionalization and epithelial-mesenchymal interactions. Mech Dev.

[12] Finch PW, Cunha GR, Rubin JS, Wong J, Ron D (1995). Pattern of keratinocyte growth factor and keratinocyte growth factor receptor expression during mouse fetal development suggests a role in mediating morphogenetic mesenchymal-epithelial interactions. Dev Dyn.

[13] Zhang X, Ibrahimi OA, Olsen SK, Umemori H, Mohammadi M, Ornitz DM (1996). Receptor specificity of the fibroblast growth factor family. The complete mammalian FGF family. J Biol Chem.

[14] Shiratori M, Oshika E, Ung LP (1996). Keratinocyte growth factor and embryonic rat lung morphogenesis. Am J Respir Cell Mol Biol.

[15] Bellusci S, Grindley J, Emoto H, Itoh N, Hogan BL (1997). Fibroblast growth factor 10 (FGF10) and branching morphogenesis in the embryonic mouse lung. Development.

[16] Miller AJ, Hill DR, Nagy MS (2018). In vitro induction and in vivo engraftment of lung bud tip progenitor cells derived from human pluripotent stem cells. Stem Cell Reports.

[17] Nogawa H, Ito T (1995). Branching morphogenesis of embryonic lung epithelium in mesenchymal-free culture. Development.

[18] Ito T, Udaka N, Nogawa H, Kitamura H, Kanisawa M (1997). Development of pulmonary neuroendocrine cells in explant culture. Lab Invest.

[19] Cardoso WV, Itoh A, Nogawa H, Mason I, Brody JS (1997). FGF-1 and FGF-7 induce distinct patterns of growth and differentiation in embryonic lung epithelium. Dev Dyn.

[20] Weaver M, Dunn NR, Hogan BL (2000). Bmp4 and Fgf10 play opposing roles during lung bud morphogenesis. Development.

[21] Barkauskas CE, Chung M-I, Fioret B, Gao X, Katsura H, Hogan BLM (2017). Lung organoids: current uses and future promise. Development.

[22] Dye BR, Hill DR, Ferguson MA (2015). In vitro generation of human pluripotent stem cell derived lung organoids. Elife.

[23] Huang SXL, Green MD, De Carvalho A (2015). The in vitro generation of lung and airway progenitor cells from human pluripotent stem cells. Nature Protoc..

[24] Chen YW, Huang SX, de Carvalho A (2017). A three-dimensional model of human lung development and disease from pluripotent stem cells. Nat Cell Biol.

[25] Miller AJ, Dye BR, Nagy MS (2017). Integrated growth factor signaling promotes lung epithelial progenitor cell expansion and maintenance in mice and human. bioRxiv.

[26] Ohtuska N, Urase K, Momoi T, Nogawa H (2001). Induction of bud formation of embryonic mouse tracheal epithelium by fibroblast growth factor plus transferrin in mesenchyme-free culture. Dev Dyn.

[27] Ryan TA (1959). Multiple comparisons in psychological research. Psychol Bull.

[28] Ryan TA (1960). Significance tests for multiple comparisons of proportions, variances, and other statistics. Psychol Bull.

[29] Ito T, Nagahara N, Ogawa T, Inayama Y, Kanisawa M (1985). Lectin binding to the luminal surface of distal airway epithelial cells of rodents. J Electron Microsc.

[30] Perl AK, Kist R, Shan Z, Scherer G, Whitsett JA (2005). Normal lung development and function after Sox9 inactivation in the respiratory epithelium. Genesis.

[31] Rockich BE, Hrycaj SM, Shih HP (2013). Sox9 plays multiple roles in the lung epithelium during branching morphogenesis. Proc Natl Acad Sci USA.

[32] Ohkubo T, Knoepfler PS, Eisenman RN, Hogan BL (2005). Nmyc plays an essential role during lung development as a dosage-sensitive regulator of progenitor cell proliferation and differentiation. Development.

[33] Butler M, Dawson M (1992). Cell culture.

[34] Platt RJ, Chen S, Zhou Y (2014). CRISPR-Cas9 knockin mice for genome editing and cancer modeling. Cell.

